# Supplementary material for: Neuroprotective Effects of Neuropeptide Y on Human Neuroblastoma SH-SY5Y Cells in Glutamate Excitotoxicity and ER Stress Conditions
Source: Cells. 2022 Nov 18;11(22):3665. doi: 10.3390/cells11223665 (PMC9688085; doi:10.3390/cells11223665)
Supplement: Supplementary file 1 [file cells-11-03665-s001.zip › cells-2009175-supplementary.pdf]

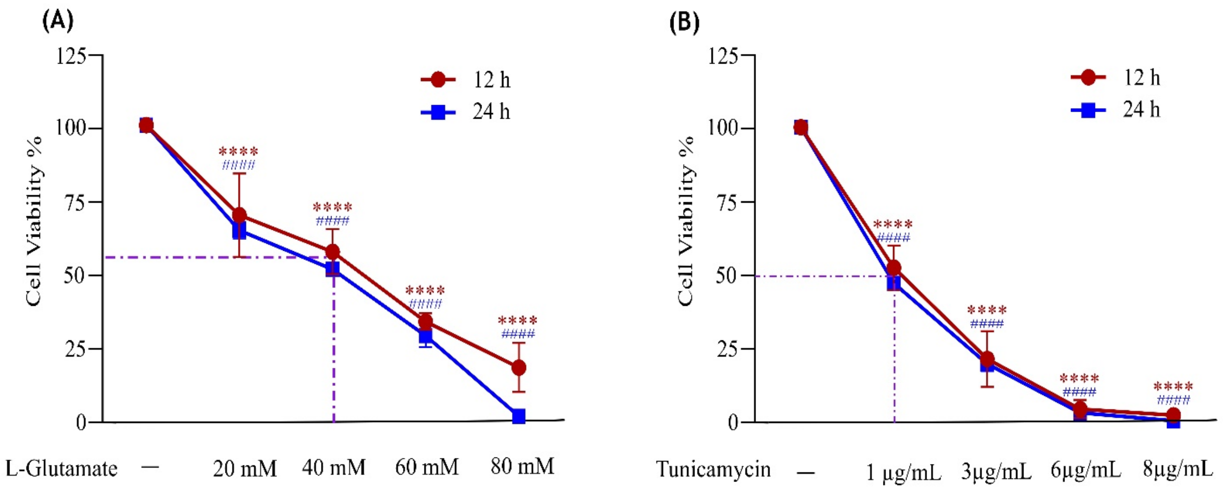

Supplementary Figure S1: Effect of various concentrations of glutamate and tunicamycin on SH-SY5Y cell survival. (A) A significant decrease in the viability in cells exposed to L-glutamate at various doses (20-80 mM) for 12 and 24 h ($F(4, 20) = 66.44$, **** $p < 0.0001$ represents significance between untreated and glutamate group in 12 h (red), #### $p < 0.0001$ in 24 h (blue), two-way ANOVA, Bonferroni's post-hoc test, n=3) and cells at 40 mM dose concentration exhibited around 50% cell viability. (B) A significant decrease in the viability in cells exposed to tunicamycin at various doses (1-8 µg/mL) for 12 and 24 h ($F(4, 20) = 91.74$, **** $p < 0.0001$ represents significance between untreated and glu-tamate group in 12 h (red), #### $p < 0.0001$ in 24 h (blue), two-way ANOVA, Bonferroni's post-hoc test, n=3) and cells at 1 µg/mL dose concentration exhibited around 50% cell viability.

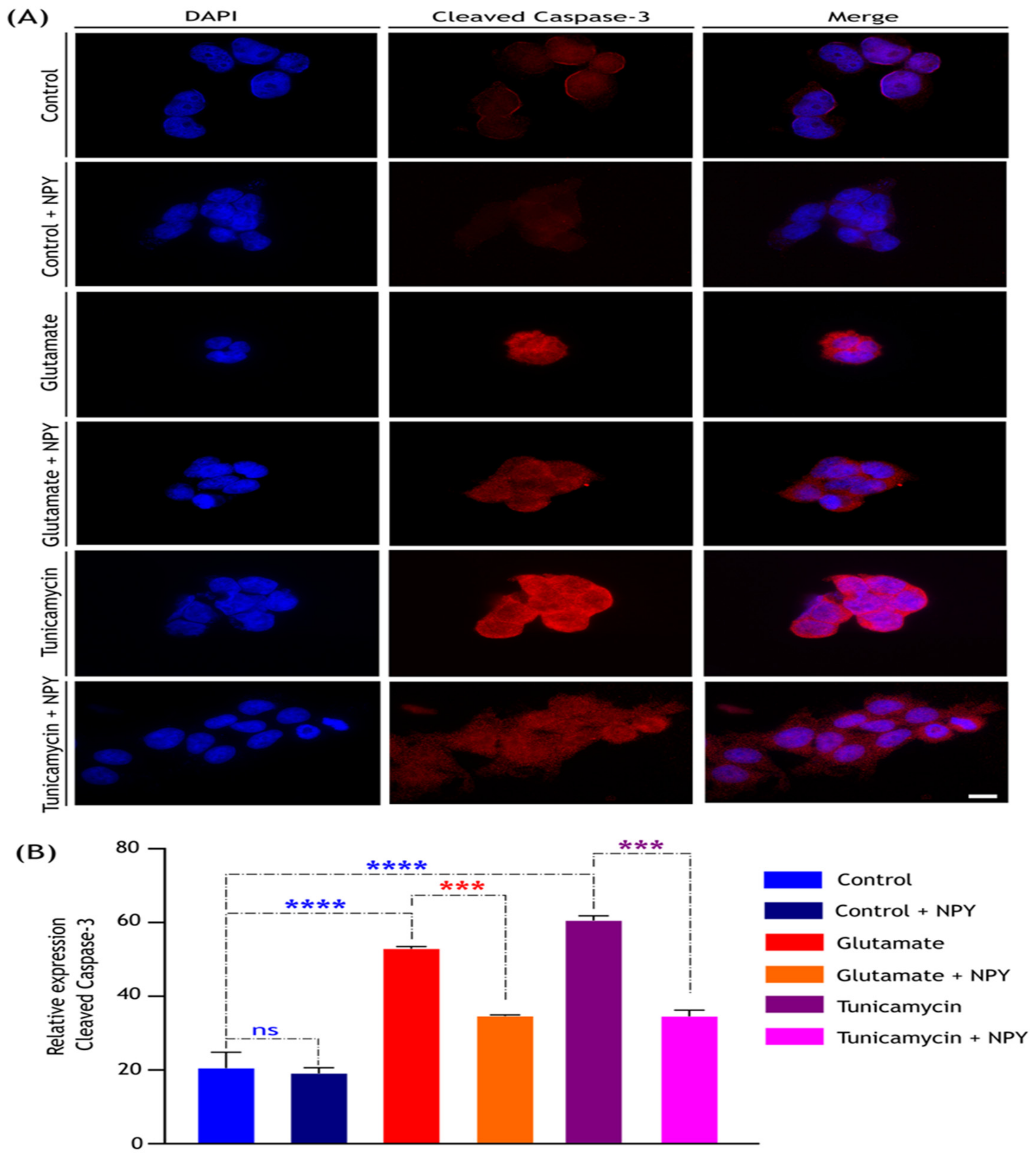

Supplementary Figure S2: NPY alleviates the apoptosis induced by glutamate and tunicamycin in SH-SY5Y cells. (A) Immunofluorescence images of treated cells showing the expression of cleaved caspase 3. After 12 h of treatment, cells were fixed and immunostained with a cleaved caspase-3 antibody (red). Nuclei were stained with DAPI (blue). Scale bar = 10 µm. (B) Cells which were exposed to toxicity (glutamate or tunicamycin) showed a significant increase in cleaved caspase-3 expression compared to control ($F(5,12)=64.42$, **** $p < 0.0001$ (blue), one way ANOVA, Bonferroni's post-hoc test, n=3) and a significant decrease in its expression was observed with NPY treatment ($F(5,12)=64.42$, *** $p < 0.001$ (red represents significance between glutamate and glutamate + NPY; purple represents significance between tunicamycin and tunicamycin + NPY), one way ANOVA, Bonferroni's post-hoc test, n=3). ns represent non-significant.

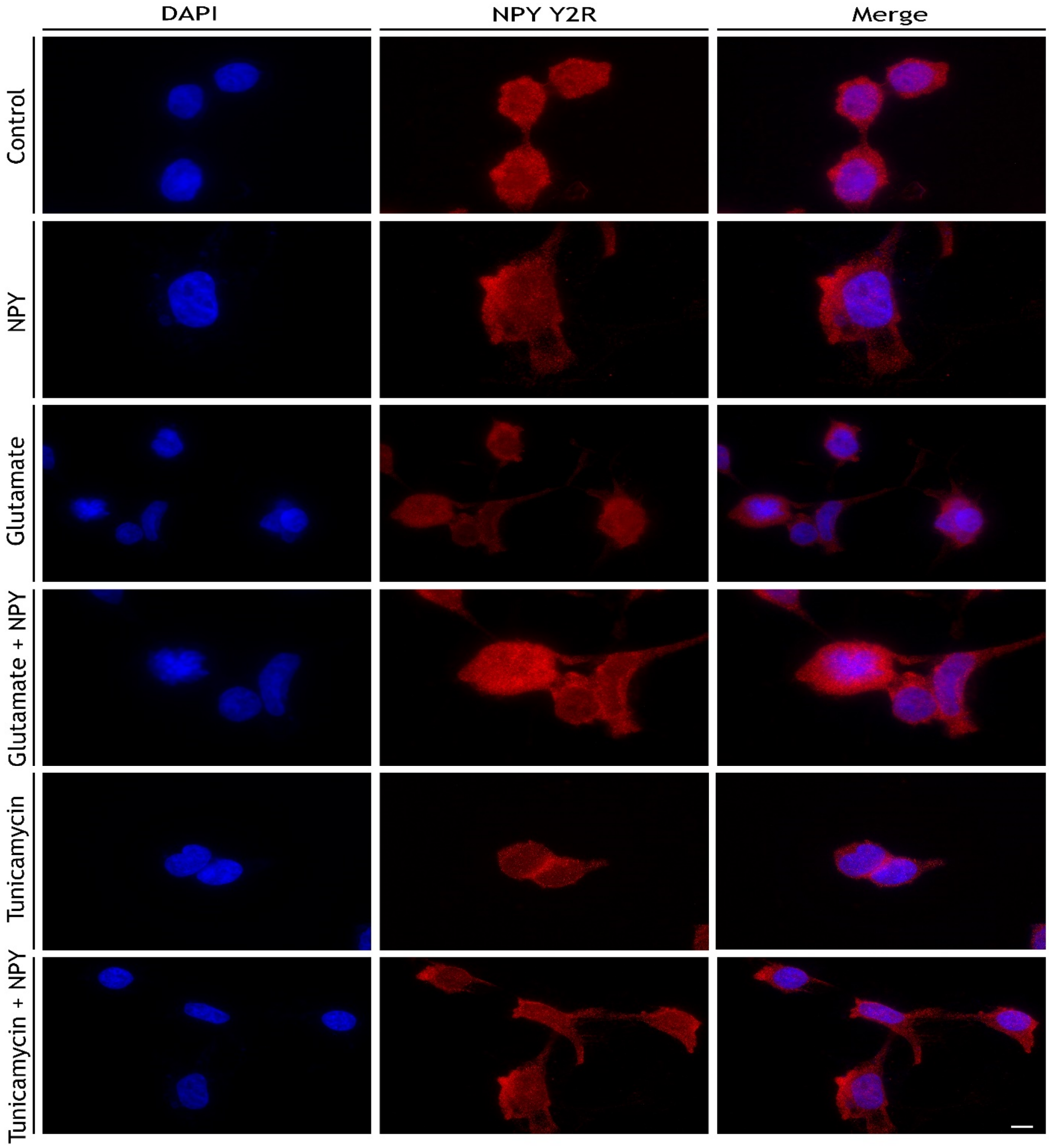

Supplementary Figure S3: Expression of NPY-Y2R in SH-SY5Y cells. Immunofluorescence images of treated SH-SY5Y cells showing the expression of NPY-Y2R. After 12 hours of treatment, cells were fixed and immunostained with an anti-NPY-Y2R antibody (red). Nuclei were stained with DAPI (blue). Scale bar = 10 μm.
